# Quality of Life among HIV Patients with NCDs Receiving Antiretroviral Therapy in Wakiso District, Uganda: Exploring Key Determinants

**DOI:** 10.4314/ahs.v25i4.6

**Published:** 2025-12

**Authors:** Christopher Ddamulira, James Wanzima, Lawrence Sserwanga, Eria Muwanguzi, Frank Pio Kiyingi, Stephen S Kizza

**Affiliations:** 1 Uganda National Council for Science and Technology, Kampala, Uganda; 2 Bugema University Graduate School, Kampala Uganda; 3 School of Post Graduate Studies and Research, Nkumba University, Entebbe, Uganda; 4 National Health Laboratories and Diagnostics Services, Ministry of Health Uganda

**Keywords:** Community, Non-Communicable Diseases, Services, Quality of Life, HIV, People Living with HIV, Uganda

## Abstract

**Background:**

Uganda, like many other nations, faces a double burden of communicable and non-communicable diseases due to the severe impacts of HIV, antiretroviral therapy, and the increasing number of HIV-positive individuals.

**Objective:**

To determine the factors that influence the quality of life among HIV patients with NCDs receiving antiretroviral therapy in Wakiso District, Uganda.

**Methods:**

A cross-sectional survey was conducted in Wakiso, Uganda, among HIV patients with NCDs receiving antiretroviral therapy and drug refills from Community Drug Distribution Points (CDDP). 219 participants completed questionnaires between November 2019 and December 2020. The factors assessed as predictors included health promotion, community support system, patient monitoring, age, sex, education level, and marital status. The diagnosis of NCDs was based on documented evidence ofiabetes Mellitus (DM) and Hypertension (HT); fasting blood sugar >7.0 mmol/L (126 mg/dl) for DM, and blood pressure > 140/90mmhg for HT. Data analysis included descriptive statistics, path analysis, and Structural Equation Modeling (SEM), which were used to verify and test the model.

**Results:**

The research found that most HIV patients were female (61.6%), aged 40 or older (94.5%), with secondary school or less education (69.4%). 54.8% were single. Community-based NCD services, such as health promotion (β=0.58, P=0.006), community support system (β=0.24, p<0.001), and patient monitoring system (β=0.46, p<0.001), directly influenced the quality-of-life improvements, along with other factors like education level (β=0.76, p<0.001) and marital status (β=0.57, P=0.002).

**Conclusion:**

The quality of life for HIV-positive individuals with NCDs in Wakiso district requires strengthening community-integrated HIV-NCD interventions to improve the overall quality of life for these individuals.

## Background

Globally, deaths from NCDs are projected to reach 52 million by 2030^31^. The deaths related to hypertension and Diabetes Mellitus among HIV patients on Antiretroviral Therapy (ART) increased due to the scale-up of the treatment and aging with HIV^14^. Most deaths occur among HIV patients with Hypertension (HT) and Diabetes Mellitus (DM) conditions. The patients need daily medication and or to make lifestyle changes to ensure they can live as well as possible with their condition^27^. Therefore, community support systems are important in reducing the burden of NCDs in People Living with HIV and AIDS.

Sub-Saharan Africa has undergone epidemiological transitions that include an epidemic of Non-Communicable Diseases (NCDs) with an estimated prevalence of hypertension of 26.5% and 12.6% for Diabetes Mellitus in South, Central, and East African regions^1^–^3^. Despite the burden of NCDs in South Africa, donor responses have primarily focused on communicable diseases^4^. Integrating global HIV/AIDS lessons into NCD programs could be more effective^5^. The successful rollout of ART has increased life expectancy, but new challenges are emerging, including NCDs in individuals on ART aging with HIV^6^. The quality of life (QoL) of people living with HIV has significantly improved due to the availability of ART and early HIV diagnosis^7^. In Sub-Saharan African countries, individuals living with HIV enjoy the same life expectancy and QoL as the general population^8^. The immunological effectiveness of the treatment has transformed HIV into a chronic condition, making health-related quality of life (HRQoL) a major focus for HIV care^9^. ART has significantly enhanced patients' physical quality of life in analyzed studies.

The National Health Policy, Ministry of Health, Uganda^10^ acknowledges the rise of non-communicable diseases (NCDs) among HIV patients on Anti-Retroviral Therapy (ART). The policy mandates a program for NCD prevention and control in public health facilities, but health workers are not providing services to community drug distribution points (CDDPs). The provision of NCD services in the communities would address sustainable development goal three, reducing premature mortality from NCDs^11^ and promoting good health by 2030^12^.

Uganda, like other sub-Saharan African countries, is grappling with a double burden of NCDs due to the impact of HIV, ART, and the population of people aging with HIV^3^,^13^. With hypertension (HT) and Diabetes Mellitus (DM) prevalences of 28.7% and 14%, Uganda has around 250,000 people with a double burden of HIV and NCD^14^. Despite existing strategies for HIV care integration^15^ in health facilities, NCDs like HT and DM have not received the attention they deserve^16^ in the CDDPs, posing a significant threat to the health system^2^,^3^,^13^. Studies reveal a link between HIV, HT, and type 2 DM due to HIV infection, long-term ART use, lifestyle, genetics, and aging^17^. DM and HT are major causes of morbidity and mortality in HIV-positive individuals, reducing life expectancy and quality of life^18^. HIV-positive individuals have a higher incidence of type 2 DM^19^. Sub-Saharan Africa faces a health crisis due to HIV and AIDS epidemics, with HT projected to rise to 125 million by 2025^20^.

Uganda has 1.5 million HIV-positive individuals, with 60% receiving lifelong antiretroviral therapy (ART) by 2016^18^. The number of patients on ART is predicted to rise to 90% due to UNAIDS 90-90-90 targets and the test and treat strategy introduced in 2016^21^. Diabetes mellitus and hypertension are NCDs that are significant for public health in Uganda. According to Kavishe^22^, the two main issues facing Uganda are the high prevalence of NCD risk factors and undiagnosed and untreated hypertension. There is a chance for prevention due to the high prevalence of NCDs that can be avoided, such as diabetes mellitus (DM). To improve chronic disease prevention, early identification, and treatment, Uganda must make significant efforts^22^.

Numerous lessons from the HIV experience can be applied to the NCD movement, as mentioned by Rabkin^23^. To address issues related to HIV and NCDs, MOH^15^ states that among the lessons learned were the need to shift responsibilities from cadres of Health Care Workers (HCW) to para-health workers in the communities, community health extension workers, VHTs, and community volunteers; to establish comprehensive clinical monitoring and assessment procedures, offer patient-centered, long-term treatment, and facilitate medication adherence.

The diversity of complex disease processes despite shared risk factors and the prolonged duration of preclinical disease necessitates screening and early identification of modifiable risk factors are among the difficulties in treating HIV and NCDs^2^. Community health workers, VHTs, and expert customers are the primary points of contact for health services; yet, they are not equipped with the skills and information required for NCD prevention, identification, and referral^24^. The quality of life for people living with HIV would therefore be improved by applying peer-led social networks to non-communicable disease prevention and management while incorporating the knowledge gained from HIV community delivery models. The existing evidence indicates that there is a burden of Non-Communicable Diseases (NCDs) among People Living with HIV (PLHIV) on Anti-Retroviral Therapy in Uganda^1^,^25^. The prevalence of NCDs such as Hypertension (28.7%) and Diabetes Mellitus (20.9%) among PLHIV on ART is increasing in Uganda^14^,^25^. There is a poor quality of life (56.4%) among PLHIV living with NCDs in Wakiso District^26^. Inadequate community-based NCD services for Hypertension and Diabetes among PLHIV on ART cause poor quality of life^27^. The World Health Organization recommends an overall good Quality of Life (QoL) among HIV patients on ART living at 60%-80% (Good) and 80%-100% (Very Good)^28^. As a result of the gaps in QoL, patients with HIV and NCDs suffer from the inability to function occupationally. Hence, understanding community-based NCD service and patient factors associated with the QoL of patients receiving ART from the communities would help to design appropriate interventions to improve their QoL.

Wakiso district in Uganda is one of the most HIV-burdened areas, with a prevalence of 10.4%^29^. In Wakiso District, 70% of ART patients receive ARVs from community drug distribution centers^24^. The district has the highest number of HIV patients receiving antiretroviral therapy in Uganda, with 47,779 people receiving ARVs from the communities^30^. However, this lack of NCD community support systems affects patients' quality of life and physical health, leading to physical issues related to HIV infection and discomfort^31^. The physical quality of life in Wakiso has been negatively impacted by the co-burden of HIV, diabetes mellitus, and hypertension in PLHIV patients on ART, resulting in physical pain, bodily malfunctions, and severe pain^14^. Therefore, the study aimed to investigate factors associated with QoL among HIV patients with NCDs receiving ART drugs from the community distribution points in Wakiso District, Uganda.

## Methods and Materials

The study employed a cross-sectional study design to identify factors associated with the quality of life for HIV-positive individuals with NCDs. All participants were adults living with HIV and NCDs receiving Antiretroviral Therapy from Community Drug Distribution Points (CDDP). The ART records of the several facilities that took part in the study were used to randomly choose the participants.

The study was carried out in the central Ugandan district of Wakiso which has the highest number of HIV patients receiving Antiretroviral Therapy from the communities^30^. The sample size was established using a formula by Krejcie and Morgan^32^. The 95% confidence interval and 5% margin of error were considered while calculating the study's sample size. The study included 219 HIV patients with NCDs (hypertension or diabetes) who were getting chronic treatment from the CDDPs in Wakiso District. The diagnosis of NCDs was based on documented evidence of DM, HT; Fasting blood sugar >7.0 mmol/L (126 mg/dl) for Diabetes Mellitus, and Blood pressure > 140/90mmhg for Hypertension. The participants for inclusion in the study were based on the hypertension and Diabetes diagnosis. The level of each patient was the analysis unit. All eligible HIV participants who were 18 years of age or older and getting ART in the comunities were asked to formally consent.

The informed consent was sought from all study participants prior to undertaking the study. The Mildmay Uganda Research Ethics Committee approved the protocol, and procedures were conducted in accordance with the Uganda National Council for Science and Technology guidelines.

The factors assessed as predictors included health promotion, community support system, patient monitoring, age, sex, education level, and marital status. A WHO standardized structured questionnaire was used to assess the predictor variables and Quality of Life (QoL) to collect data^33^. To compute Cronbach's alpha, the tool was piloted, and data was coded on the fifty items that were scored in the instrument and entered SPSS, then Cronbach's alpha coefficients analysis was done to derive the Cronbach's alpha for the instrument as 0.867. A minimum acceptance composite reliability measure of Cronbach's alpha (0.7) was considered a reasonable measure of the internal reliability of the questionnaire as recommended by Amin and Tavakol^34^,^35^. Utilizing the structural equation modeling (SEM) technique, Amos software was used for inferential statistics, and SPSS Version 20 was used for descriptive data analysis.

## Results

Sixty-one percent of the responses were women. 94.5% of the participants were over 40 years. Based on the respondents' educational attainment, 69.4% had less than a secondary education, and 41.1% were married.

Table 2 revealed that factors such as the patient monitoring system (β = 0.46, p<0.001), community support system (β = 0.24, p<0.001), and health promotion (β = 0.58, P = 0.006), were associated with quality of life. Overall, community-based services factors were significantly associated with the participants' quality of life (QoL). In a similar vein, the study participants' marital status (β = 0.57, P = 0.002) and level of education (β = 0.76, p<0.001) were significantly associated with quality of life. It suggested that raising community levels of education and getting married were key components associated with the QoL. Family elements, such as partner engagement, can significantly promote medicine compliance and foster a calm environment, both of which are critical for managing hypertension and diabetes.

**Table 2:** Structural Equation Modelling (SEM) for Factors Associated with QoL of the participants

Variables	Coefficient (β)	Standard Error (S.E)	p-value	95% Conf. Interval	

				Lower	Upper
**Predictors of QoL**					
Health Promotion	0.58	0.070	0.006**	0.057	0.633
Community Support System	0.24	0.058	0.000**	0.129	0.355
Patient Monitoring System	0.46	0.059	0.000**	0.147	0.578
Sex	0.059	0.091	0.513	0.119	0.237
Marital Status	0.58	0.095	0.002**	0.291	0.581
Education Level	0.76	0.059	0.000**	0.191	0.839
Age in years	-0.0023	0.005	0.619	-0.011	0.007

**Source: Primary data, 2019**

Table 3 shows the overall indicators that the model fitted perfectly well; Root Mean Squared Error of Approximation (RMSEA) = 0.000, Comparative Fit Index (CFI) = 1.000, Tucker-Lewis Index (TFI) =1.000). It is asserted that RMSEA values range from 0 to 1, < 1 indicates a better model fit; CFI & TLI values range from 0 to 1, > 0.95 indicates a better fit^36^. Likewise, SRMSR values <0.08 generally indicate adequate fit. This meant that the community-based services factors and patient factors (marital status and education level) were associated with the QoL.

**Table 3:** Goodness of Fit Results for the Model

Test	Result	Interpretation
Root Mean Squared Error of Approximation (RMSEA)	0.000 (95%CI: 0.000-0.000)	Very Good Fit
Pclose	1.000, <= 0.05	
Comparative Fit Index (CFI)	1.000	Very Good Fit
Tucker-Lewis Index (TFI)	1.000	Good Model Fit
Standardized Root Mean Squared Residual (SRMSR)	0.000	Perfect Fit
Coefficient of determination (CD)	0.038	

**Source: Primary data, 2019**

## Discussion of the Results

The study found a significant association between individuals' educational attainment, marital status, health promotion, community support and patient support systems and the patient QoL. Prior UNAIDS^37^ studies showed positive outcomes for long-term treatment in the community. Establishing community-based NCD services could be a crucial strategy for managing and preventing NCDs among HIV-positive individuals living with NCDs, adapting to local contexts and cultures in Uganda. Inconclusive results were found in systematic reviews that examined the quality of life (QoL) of populations infected with HIV and were based on various therapies and observational studies^38^. The results demonstrated that substantial improvement of the community-based factors towards the patients with HIV and NCDs would enhance their overall quality of life.

Furthermore, research by Bhatta and Liabsuetrakul^39^ showed that community-integrated HIV-NCD interventions in Nepal improved the QoL for HIV patients through community support systems and health promotion activities. Additionally, the empowerment intervention, which employed health promotion initiatives and benefited from community systems structures, effectively raised the quality of life for HIV-positive individuals in Nepal^39^.

In a similar vein, the Decroo^40^ cohort study in Tete Province, Mozambique, found that community ART groups in the community had higher rates of patient retention and better clinical outcomes than one-on-one care. The study suggests that patient-driven community HIV and NCD distribution groups, involving lay cadres, knowledgeable clients, and community volunteers, can enhance patient quality of life and retention among ART patients with NCDS in communities. The QoL of HIV-positive populations was the topic of systematic reviews based on various therapies and observational studies, but the results were inconclusive^38^.

The study found that married individuals with higher education levels had easier access to support networks than single individuals with lower education levels. This aligns with cross-sectional research in India, which found married individuals with post-secondary education degrees use community support networks to improve their QoL^41^,^42^. Age and sex did not significantly impact QoL. Moores^9^,^43^ research found age and married status as significant determinants of community NCD services uptake and quality of life improvement. However, Ha and Joshi^41^,^42^ in the earlier studies compared chronic illness risk factors among participants over 40 years old, as all participants had non-communicable diseases (NCDs) and had an average age of 56. Therefore, improving the literacy levels of the communities through formal education attainment is an important factor for the prevention and uptake of the NCD preventive and management services in the communities. It implied that the higher the level of education of the individuals in the communities facilitated, the more uptake of the NCD services that improved the patient's outcomes.

Additionally, research conducted in China revealed that men were more likely than women to acquire hypertension and diabetes mellitus (DM)^44^,^45^. Because of this, there was no comparison group in the trial and every participant had an NCD. Thus, it clarified why there were no discernible associations between the moderating variables (sex and age) and QoL in Table 2.

## Conclusion and Recommendations

### Conclusion

The study found that patient and community-based NCD factors would significantly improve the QoL for HIV-positive individuals living with NCDs in community settings.

### Recommendations

The government and District Health Officers should enhance community-integrated HIV-NCD interventions in Wakiso district, focusing on health promotion strategies, community support structures, and patient monitoring systems for HIV and AIDS patients, with empowerment interventions by the community systems structures.

## Figures and Tables

**Table 1:** Demographic Characteristics of Respondents

Variables	Frequency (N)	Percentage (%)
**Sex**		
Male	75	34.2
Female	135	61.6
**Age**		
Below 40 years	3	1.4
40 years and above	207	94.5
**Education Level**		
Below Secondary	152	69.4
Secondary and above	58	26.5
**Marital Status**		
Unmarried	120	54.8
Married	90	41.1
